# Antidiabetic effects of *Brucea javanica* seeds in type 2 diabetic rats

**DOI:** 10.1186/s12906-017-1610-x

**Published:** 2017-02-06

**Authors:** Abdulwali Ablat, Mohammed Farouq Halabi, Jamaludin Mohamad, Muhammad Hafiz Husna Hasnan, Hazrina Hazni, Ser-huy Teh, Jamil A. Shilpi, Zulqarnain Mohamed, Khalijah Awang

**Affiliations:** 1 Institute of Biological Science, Faculty of Science, University of Malaya, 50603 Kuala Lumpur, Malaysia; 2 Department of Biomedical Science, Faculty of Medicine, University of Malaya, 50603 Kuala Lumpur, Malaysia; 3 Department of Chemistry, Faculty of Science, University of Malaya, 50603 Kuala Lumpur, Malaysia; 4 Center for Natural Products and Drug discovery (CENAR), University of Malaya, 50603 Kuala Lumpur, Malaysia; 5 Genetics and Molecular Biology Unit, Institute of Biological Science, Faculty of Science, University of Malaya, 50603 Kuala Lumpur, Malaysia; 6 Department of Biology, Faculty of Sciences & Art, Taibah University, Al-Ula, Saudi Arabia; 7 Pharmacy Discipline, Khulna University, Khulna, 9208 Bangladesh

**Keywords:** *Brucea javanica*, T2D, α-glucosidase, GP-α, Cytokines, Luteolin

## Abstract

**Background:**

*Brucea javanica* (*B. javanica*) seeds, also known as “*Melada pahit*” in Indo-Malay region are traditionally used to treat diabetes. The objective of this study was to determine antidiabetic, antioxidant and anti-inflammatory effects of *B. javanica* seeds on nicotinamide (NA)-streptozotocin (STZ) induced type 2 diabetic (T2D) rats and to analyze its chemical composition that correlate with their pharmacological activities.

**Methods:**

A hydroethanolic extract of *B. javanica* seeds was fractionated with n-hexane, chloroform and ethyl acetate. An active fraction was selected after screening for its ability to inhibit α-glucosidase and glycogen phosphorylase α (GP-α). Isolation and characterization were carried out by using column chromatography, NMR and LCMS/MS. All isolates were assayed for inhibition of GP-α and α-glucosidase. Antidiabetic effect of active fraction was further evaluated in T2D rat model. Blood glucose and body weight were measured weekly. Serum insulin, lipid profile, renal function, liver glycogen and biomarkers of oxidative stress and inflammation were analyzed after 4-week treatment and compared with standard drug glibenclamide.

**Results:**

Ethyl acetate fraction (EAF) exerted good inhibitory potential for α-glucosidase and GP-α compared with other fractions. Chromatographic isolation of the EAF led to the identification of seven compounds: vanillic acid (1), bruceine D (2), bruceine E (3), parahydroxybenzoic acid (4), luteolin (5), protocatechuic acid (6), and gallic acid (7). Among them, Compound (5) was identified as the most potent inhibitor of GP-α and α-glucosidase and its GP-α inhibitory activity (IC_50_ = 45.08 μM) was 10-fold higher than that of caffeine (IC_50_ = 457.34 μM), and α-glucosidase inhibitory activity (IC_50_ = 26.41 μM) was 5.5-fold higher than that of acarbose (IC_50_ = 145.83 μM), respectively. Compounds (4), (6), and (7) inhibited GP-α activity in a concentration-dependent manner with IC_50_ values of 357.88, 297.37, and 214.38 μM, and their inhibitory effect was higher than that of caffeine. These compounds exhibited weak potency on α-glucosidase compared with acarbose. Compounds (1), (2), and (3) showed no inhibition on both GP-α and α-glucosidase. In vivo study showed that EAF treatment significantly reduced blood glucose level, increased insulin and glycogen contents, decreased markers of oxidative stress and inflammation, and lipid levels in T2D rats compared with untreated group.

**Conclusions:**

The EAF has potential therapeutic value for the treatment of T2D via acting as GP-α and α-glucosidase inhibitors by improving hepatic glucose and carbohydrate metabolism, suppressing oxidative stress, and preventing inflammation in T2D rats. According to the results, the efficacy of EAF could be due to the presence of luteolin along with synergistic effect of multiple compounds such as parahydroxybenzoic acid, protocatechuic acid, and gallic acid in *B. javanica* seeds.

## Background

Type 2 diabetes (T2D) is a metabolic dysfunction characterized by hyperglycemia resulting from insulin resistant and β-cell dysfunction. T2D affected 382 million people globally in 2013, and it is estimated to rise up to 592 million by 2035 [1]. The current therapy of T2D aims to control hyperglycemia within normal level (between 4.4 to 7.2 mmol/L) and prevent the progression of its related complications [2]. Strategies to control T2D can be achieved by use of oral agents that acts with different mechanisms such as insulin secretagogues and sensitizers [3], inhibitors of dipeptidyl peptidase IV (DPP-IV) [4], α-glucosidase [5], SGLT2 [6], and glycogen phosphorylase α (GP-α). Monotherapy or combination treatments with oral agents improve management of hyperglycemia in the early stage of therapy, but fail to reach target glycemic control in long-term [7–9]. Other ways of controlling T2D is pancreas and islet transplantation. The evidence showed that the function of β-cell is progressively declined after a few years of islet transplantation. As a result, most diabetic patients have to revert to oral anti-diabetic agents and insulin treatment, or a combination of both within a few years [10].

Several studies reported that oxidative stress has a direct link to the pathogens of diabetes that lead to insulin resistant, β-cell dysfunction, and impaired glucose tolerance in hyperglycemic subjects [11, 12]. It is also directly linked to protein damage and diabetic vascular complications such as cardiomyopathy, retinopathy, nephropathy, and neuropathy [13, 14]. Among them, diabetic cardiomyopathy was found to be associated with increased accumulation of ROS in T2D [15]. It was also reported that oxidative stress is involved in cytokine-mediated inflammation in T2D [16]. Therefore, potential therapeutic agents that improve the efficiency in controlling T2D and retard its related complications are urgently needed.

The genus Brucea consisting of six species is a member of the Simaroubaceae family and believed to have originated in tropical Africa and tropical Asia. It is a dominant species in this genus, and most commonly found in Malaya Peninsula [17]. The compounds isolated from this species showed wide spectrum of biological effects [18] and gained increasing interest for further study. Bruceine D and E, isolated from *B. javanica* seeds, exhibited blood glucose lowering effect in both nondiabetic mice and STZ-induced diabetic rats at lower dose (1 mg/kg b.w.) during 0-8 h screening [19]. However, chemical entities responsible for potential inhibitory effect of this plant against GP-α and α-glucosidase enzymes are yet to be identified. Therefore, this study reports the effect of fractions from *B. javanica* seed extracts for GP-α and α-glucosidase inhibition to select the most potent inhibitor and evaluates antihyperglycemic, anti-inflammatory, and antioxidant activities of active fraction in T2D rats.

## Methods

### Chemicals

Nicotinamide (NA), streptozotocin (STZ), p-nitrophenyl α-D-glucopyranoside (*p*NPG), and α-glucosidase were purchased from Sigma-Aldrich®, St. Louis, MO, USA. Rat TNF-α, rat IL-6, and rat IL-1β ELISA kits were purchased from eBioscience (San Diego, CA USA). Rat insulin ELISA kit was purchased from Mercodia AB (Uppsala, Sweden). TBARS, Glutathione, and Glycogen assay kits were purchased from Cayman chemical company (Ann Arbor, MI, USA). All of the other chemicals are analytical grade purchased from Sigma and Merck Chemical Co.

### Sample collection, extraction and fractionation

The seeds of wild grown *B. javanica* were collected from Bukit Tampin Reserved Forest (2.495 N, 102.201E), Tampin, Negeri Sembilan, Malaysia, during the month of November. Botanical identification of the sample was performed by comparing with a voucher specimen available in the University of Malaya botanical garden and it was further confirmed by Teo Leong Eng from Department of Chemistry, Faculty of Science, University of Malaya. A voucher specimen (KL5794) was kept at the herbarium, faculty of Science, University of Malaya. The seeds were dried in an oven and grinded. The powdered *B. javanica* seed was extracted with 95% ethanol and partitioned with solvents of different polarities as described previously [20].

### GP-α and α-glucosidase inhibition assay

The fractions from *B. javanica* seeds were evaluated as GP-α inhibitors [20] and were further tested for their α-glucosidase inhibitory activity as described previously [21]. Briefly, 50 μL of samples or standard were mixed with 100 μL of α-glucosidase (0.1 U/mL) in phosphate buffer (0.1 M, pH 6.9) and incubated at 37 °C for 10 min. The reactions were initiated by addition of *p*-Nitrophenyl α-D-Glucoside (*p-*NPG, 50 μL) in phosphate buffer (0.1 M, pH 6.9) and incubated again at 37 °C for 30 min. The reactions were terminated using NaCO_3_ (1 M, 50 μL). The *p-*nitrophenol released from *p-*NPG in the presence of α-glucosidase was detected at 405 nm using microplate reader (Sunrise, Austria). Phosphate buffer (50 μL) was used as control. Blank readings (without substrate) were subtracted from specific sample wells and the percentage of α-Glucosidase inhibition (αGI) was calculated as following formula: αGI (%) = [(A_control_ – A_sample_)/A_control_] × 100 [22].

### Isolation and structure determination

#### Column chromatography

The EAF (7.6 g) was loaded onto a column packed with silica gel (0.40-0.63 μM, Merck Germany). Gradient elution was performed using *n*-hexane and DCM (90:10–0:100) followed by DCM and MeOH (100:0–70:30) as mobile phase. All fractions were concentrated, monitored by TLC, and visualized under UV light. Total 91 fractions were collected and the fractions with similar R_f_ values were pooled together to afford twelve fractions (F1-F12). F1 appeared as white crystals which showed a single spot on TLC and yield compound 1 (12 mg). F7 and F9 identified as light yellow powder to yield compound 2 (45 mg) and 3 (56 mg). F3 was subjected to Sephadex LH20 column, eluted with DCM and MeOH (65:35 – 55:45) to get 40 fractions. Fractions 29–35 were combined and dried to get compound 4 (199 mg). Sephadex LH20 column of F5, eluted with DCM and MeOH (65:35 – 45:55), yielded 35 fractions. Fractions 22–27 were combined to get compound 5 (9 mg). F6 was fractionated in the same fashion as above to afford 36 fractions. Fraction 27 showed a single spot on TLC and dried to yield compound 6 (53.8 mg). Sephadex LH20 column of F8 using DCM and MeOH (60:40 – 20:80) yielded 50 fractions. Upon drying, fraction 20 gave rise to crystals showing single spot on TLC and designated as compound 7 (7.6 mg).

#### Nuclear magnetic resonance (NMR)

1D- (^1^H, ^13^C & DEPT) and 2D-NMR (COSY, HSQC, HMBC, NOESY) spectra were recorded in deuterated pyridine (C_5_D_5_N) and deuterated methanol (CD_3_OD) using Bruker Avance III 400 NMR spectrometer. Chemical shifts (δ) were expressed in ppm and coupling constants (*J*) were given in Hz.

#### HPLC-MS analysis

The HPLC-MS system consisted of an Agilent 6530 Q-TOF MS equipped with Dual AJS ESI as the ion source and coupled to an Agilent 1200 HPLC system. The HPLC system was equipped with a binary pump, an auto plate-sampler, and a thermostatically controlled column compartment. Chromatographic separation was carried out using an Agilent Zorbax Eclipse Plus C18 column Rapid Resolution HT (2.1 × 1 mm, 1.8 μm). The mobile phase (solvent A, 0.1% formic acid in water; solvent B, 0.1% formic acid in ACN) was eluted at a flow rate of 0.5 mL/min. The elution was gradient (0 min, 90% A, 1 min, 90% A, 20 min, 50% A, 24 min, 50% A, 25 min, 90% A, 30 min, 90% A). The total run time was 30 min, and the injection volume was 10 μL.

### Determination of GP-α and α-glucosidase inhibition activities of isolated compounds

α-glucosidase inhibition activity of isolated compounds were determined according to the method described in section 2.3 and GP-α inhibition activity was measured according to our previously reported method [20].

### In vivo anti-diabetic activity

#### Experimental animals

Sprague Dawley (SD) rats of both sexes were obtained from Animal Experimental Unit of the University of Malaya where they were bred and housed according to Guide for the Care and Use of Laboratory Animals of the National Institutes of Health (USA). The animal protocol performed in this study was approved by the Institutional Animal Care and Use Committee at the University of Malaya. Ethic No: ISB/23/05/2013/AA (R).

Total 30 SD rats (eight-week-old, 200–230 g) were dived into 5 groups (*n* = 6, 3 male and 3 female in each group) and were housed in cages under standard laboratory conditions with a12-h dark–light cycle and humidity-controlled environment with a room temperature of 22 ± 3 °C and relative humidity of 65 ± 5%. The rats were allowed access to Laboratory Rodent Chow and drinking water ad libitum and were received human care according to the guidelines.

#### Induction of type 2 diabetes (T2D)

The experimental rats were fasted overnight (16 h) and diabetes was induced by single intraperitoneal (i.p) injection of STZ (60 mg/kg b.w.) freshly prepared in 0.1 M citrate buffer (pH 4.5) 15 min after i.p injection of NA (100 mg/kg b.w.) dissolved in normal saline. Diabetes was confirmed 3 weeks after NA-STZ induction by measuring tail vein blood glucose levels using glucose meter (Accu-check Performa, Rochi diagnostic, USA). The rats having blood glucose levels higher than 11 mmol/L were considered as diabetic and selected for study [23].

#### Treatment protocol

The experimental rats were divided into 5 groups (*n* = 6; 3 M/3 F) as following. Group I: non-diabetic control (NDC) and Group II: diabetic control (DC) consisted of rats were allowed to free access of water; Group III and IV were treated orally with EAF (25 and 50 mg/kg/day b.w.) diluted in distilled water, Group V was treated orally with Glibenclamide (10 mg/kg/day b.w.) and served as standard drug. All groups except group 1 are diabetic. The selected doses (25 and 50 mg/kg/day b.w) were based on prior acute oral toxicity study [20].

#### Determination of fasting blood glucose levels and body weights

After diabetes was confirmed, rats were divided into specific groups which were mentioned in the section of treatment protocol above. Fasting blood glucose (FBG) levels and body weight of rats were measured and it was considered as 0 day. The extracts and standard drug were administered orally on a daily base in single dose for 28 days. At the end of each week, animals were fasted overnight and body weights were recorded using electronic balance. Blood samples were obtained from the tail vein of the rats by Accu-Chek FastClix lancing device and blood glucose levels were analysed using glucose meter (Accu-check, Roche Diagnostics, USA).

#### Oral glucose tolerance test (OGTT) in experimental rats

On the 25^th^ day of treatment, the OGTT was carried out according to the previously reported method [20]. All animals were fasted overnight (16 h) before commencing the experiments. Group I (non-diabetic control) and Group II (diabetic control) weretreated with distilled water, Group III and Group IV were given EAF (25 and 50 mg/kg b.w), and Group V was given glibenclamide (10 mg/kg b.w.) using oral gavage, respectively. After 30 min, α-D-glucose (2 g/kg b.w.) was administered orally into all groups of rats. Blood samples were collected from the tail vein at 0 (immediately after glucose load), 30, 60, 90, and 120 min, and blood glucose levels were determined by glucose oxidase method using a commercial glucose meter (Roche, USA). Total glycemic responses to OGTT were calculated from respective areas under the curve for glucose (*AUC*_glucose_) by trapezoid rule for the 120 min.

$$ {\mathrm{AUC}}_{\mathrm{glucose}}=\frac{{\mathrm{C}}_1 + {\mathrm{C}}_2}{2} \times \left({\mathrm{t}}_2 - {\mathrm{t}}_1\right) $$

Where, C_1_ and C_2_ are concentrations of glucose at different time points; t_1_ and t_2_ are different tested time points.

#### Collection of serum and tissue samples

At the end of study period, rats were fasted overnight, anesthetized, and the blood sample was collected by cardiac puncture. At the end, the rats were sacrificed by cervical dislocation. The blood sample was centrifuged 2000 x g for 15 minute at 4 °C, serum was collected and stored at −80 °C until analyses. The liver was carefully removed, washed in ice-cold phosphate buffered saline (PBS, pH 7.4) to remove the blood. A piece of liver sample was rinsed in liquid nitrogen, stored at −80 °C for glycogen assay.

#### Determination of serum insulin levels

The serum insulin levels were quantified by using rat insulin ELISA kit (Mercodia AB, Uppsala, Sweden) according to the manufacturer’s instructions. Insulin resistance (IR) was calculated according to the homeostasis model assessment by using the following equation [24]

$$ \mathrm{HOMA}-\mathrm{I}\mathrm{R}=\left[\mathrm{glucose}\left(\mathrm{mmol}/\mathrm{L}\right)\times \mathrm{insulin}\left(\upmu \mathrm{U}/\mathrm{mL}\right)\right]/22.5 $$

#### Measurement of serum lipid profiles, renal, and liver functions

The levels of serum total cholesterol (TC), triglycerides (TG), low density lipoprotein cholesterol (LDL-C), and high density lipoprotein cholesterol (HDL-C), total protein, alkaline phosphatase (ALP), alanine aminotransferase (ALT), aspartate aminotransferase (AST), urea, creatinine were analyzed using automatic biochemical analyzer (Beckman-700, Fullerton, CA, USA)

#### Determination of MDA and GSH levels in the serum

The malondialdehyde (MDA), a byproduct of lipid peroxidation, and glutathione (GSH), a marker of antioxidant defense, were quantified by commercial kit (Cayman chemical company, Ann Arbor, MI, USA) according to the manufacturer’s instructions.

#### Determination of serum cytokine levels

The quantification of cytokine in the serum was assessed by enzyme-linked immunosorbent assay (ELISA) using commercial kits for rat TNF-α, rat IL-6, and rat IL-1β (eBioscience, San Diego, CA USA) according to the manufacturer’s instructions.

#### Measurement of liver glycogen contents

The glycogen contents in the liver were measured using a glycogen assay kit (Ann Arbor, MI, USA) according to the manufacturer’s instructions.

#### Statistical analysis

The results are expressed as mean ± standard error (M ± SE) for six rats in each group. Statistical analysis was performed using SPSS (16.0) software. The differences between treated and untreated groups were assessed by analysis of variance (ANOVA), followed by Tukey’s multiple comparison test. Statistical significance was defined as *p* < 0.05.

## Results

### GP-α and α-glucosidase inhibition activity

**Fig. 1:**
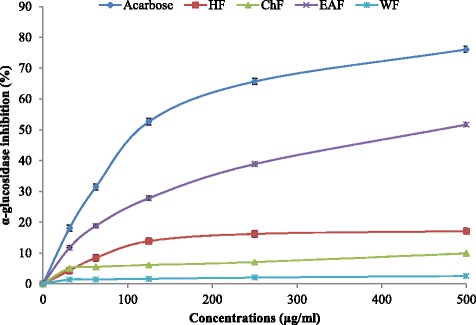
α-glucosidase inhibition by *B. javanica* seed fractions. HF: hexane fraction; CHF: chloroform fraction; EAF: ethyl acetate fraction; WF, water fraction. The values are shown in mean ± SE (*n* = 3)

GP-α inhibitory EAF fraction [20] showed the best inhibition of α-glucosidase compared to other fractions. As shown in Fig. 1, the EAF had the strongest α-glucosidase inhibition effect with IC_50_ value of 483.93 μg/ml, whereas HF, ChF, and WF showed maximal inhibition of 17.07%, 9.89%, and 2.53% at the highest concentration tested, respectively. In comparison, standard drug acarbose inhibited α-glucosidase activity (IC_50_ = 118.73 μg/ml) and its potency was 4-fold higher than that of EAF, respectively.

### Characterization of isolated compounds from EAF by LCMS and NMR

**Fig. 2:**
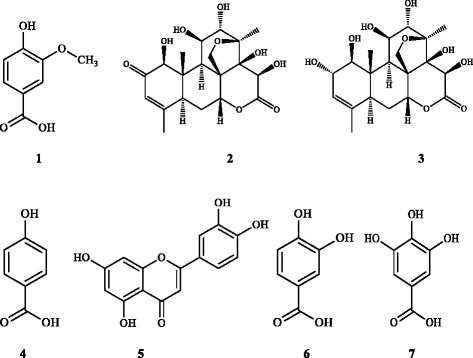
The structure of isolated compounds from EAF of *B. javanica* seeds

Phytochemical investigation of the bioactive fraction (EAF) led to the isolation of seven compounds namely, vanillic acid, bruceine D, bruceine E, para-hydroxybenzoic acid, luteolin, protocatechuic acid and gallic acid (Fig. 2). Among them, bruceine D, bruceine E, and para-hydroxybenzoic acid were identified as the major constituents of EAF. The structures of the isolated compounds were identified by a combination of mass spectrometry and extensive 1D and 2D NMR experiments. It was further confirmed by comparison of their spectroscopic data with those reported in the literature.

#### Vanillic acid (1)

Compound 1 was isolated as colorless needle. Its molecular formula of C_8_H_8_O_4_ was determined by HRESIMS ion peak at *m/z* 167.09 [M-H]^−^ (calcd for C_8_H_7_O_4_, 167.03). ^1^H NMR (400 MHz, CD_3_OD): *δ* 3.91 (3H, *s*, OCH_3_-8), 4.90 (1H*, brs*, H-4), 6.85 (1H, *d*, H-5), 7.57 (1H*, brs*, H-2), 7.57 (1H*, brs*, H-6). ^13^C NMR (100 MHz, CD_3_OD): *δ* 55.0 (C-8), 112.4 (C-2), 114.4 (C-5), 121.7 (C-1), 123.9 (C-6), 147.3 (C-3), 151.3 (C-4), 168.8 (C-7).

#### Bruceine D (2)

Compound 2 was isolated as white amorphous powder. Its molecular formula was determined to be C_20_H_26_O_9_ on the base of its HRESIMS ion peak at *m/z* 411.3018 [M + H]^+^ (calcd for C_20_H_27_O_9_, 411.1655). See Table 1 for ^1^H NMR (400 MHz, CD_3_OD) and ^13^C NMR (100 MHz, CD_3_OD).

**Table 1:** ^1^H (400 MHz) and ^13^C (100 MHz) NMR data of bruceine D in CD_3_OD

Position	Bruceine D		Bruceine E	
	δ_H_	δ_C_	δ_H_	δ_C_
1	4.26 (1H, *s*)	81.6	3.54 (1H, *d* 7.3)	81.4
2	-	198.5	4.01 (1H, *dd* 1.3, 7.3)	72.8
3	6.05 (1H, *s*)	123.8	5.41 (1H, *d* 1.3)	123.8
4	-	164.3	-	135.3
5	2.96 (1H, *d*)	43.0	2.42 (1H, *d* 12.8)	42.4
6	2.38 (1H, *dt*), 1.85 (1H, *td*)	27.3	2.17 (1H, *dt* 2.8,), 1.70 (1H, *td*)	27.2
7	5.12 (1H, *t*)	79.8	5.08 (1H, *t* 2.7)	80.6
8	-	49.3	-	49.6
9	2.42 (1H, *d*)	44.8	2.08 (1H, *d* 4.2)	45.9
10	-	44.8	-	44.0
11	4.60 (1H, *d*)	74.1	4.60 (1H, *d* 4.4)	74.4
12	3.78 (1H, *brs*)	80.0	3.76 (1H, *brs*)	79.8
13	-	83.6	-	83.4
14	-	81.0	-	81.0
15	5.24 (1H, *s*)	69.3	5.15 (1H, *s*)	69.2
16	-	174.9	-	175.0
18	1.99 (3H, *s*)	21.2	1.67 (3H, *s*)	19.7
19	1.19 (3H, *s*)	10.1	1.24 3H, *s*)	10.8
20	4.54 (1H, *d*), 3.84 (1H, *d*)	69.0	4.63 (1H, *d* 7.3), 3.83 (1H, *d* 7.3)	69.4
21	1.44 (3H, *s*)	17.1	1.43 (3H, *s*)	17.0

Chemical shifts are in ppm. Coupling constants in the parentheses are in Hz

#### Bruceine E (3)

Compound 3, a white, amorphous powder, gave a molecular of C_20_H_28_O_9_ as determined by HRESIMS ion peak at *m/z* 435.3028 [M + Na]^+^ (calcd for C_20_H_28_O_9_Na, 435.1631). See Table 1 for ^1^H NMR (400 MHz, CD_3_OD) and ^13^C NMR (100 MHz, CD_3_OD).

#### Para-hydroxybenzoic acid (4)

Compound 4, a white, amorphous powder, gave a molecular of C_7_H_6_O_3_ as determined by HRESIMS ion peak at m/z 137.00 [M-H]^−^ (calcd for C_7_H_5_O_3_, 137.02). ^1^H NMR (400 MHz, CD_3_OD): *δ* 7.90 (2H*, d*, *J* = 14 Hz, H-2, H-6), 6.83 (2H, *d*, *J* = 14 Hz, H-3, H-5). ^13^C NMR (100 MHz, CD_3_OD): *δ* 114.6 (C-3, C-5), 121.3 (C-1), 131.6 (C-2, C-6), 162.0 (C-4), 168.7 (C-7).

#### Luteolin (5)

Compound 5 was isolated as a yellow, amorphous powder. Its molecular formula was determined to be C_15_H_10_O_6_ on the base of its HRESIMS ion peak at *m/z* 285.10 [M-H]^−^ (calcd for C_15_H_9_O_6_, 285.04). ^1^H NMR (400 MHz, CD_3_OD): *δ* 6.23 (1H*, s*, H-6), 6.46 (1H, *s*, H-8), 6.56 (1H, *s*, H-3), 6.92 (1H*, d*, *J* = 8.4 Hz, H-5′), 7.39 (1H*, s*, H-2′), 7.40 (1H*, d*, *J* = 8.4 Hz, H-6′). ^13^C NMR (100 MHz, CD_3_OD): *δ* 93.6 (C-8), 98.7 (C-6), 102.5 (C-3), 103.9 (C-10), 112.8 (C-2′), 115.4 (C-5′), 118.9 (C-6′), 122.3 (C-1′), 145.6 (C-3′), 149.6 (C-4′), 158.0 (C-5), 161.8 (C-9), 164.4 (C-2), 165.0 (C-7), 182.5 (C-4).

#### Protocatechuic acid (6)

Compound 6, a white, amorphous powder, gave a molecular of C_7_H_6_O_4_ as determined by HRESIMS ion peak at *m/z* 153.00 [M-H]^−^ (calcd for C_7_H_5_O_4_, 153.01). ^1^H NMR (400 MHz, CD_3_OD): *δ* 6.82 (1H*, d*, *J* = 8.2 Hz, H-5), 7.45 (1H, *dd*, *J* = 14 Hz, 2.0 Hz, H-6).7.46 (1H*, d*, *J* = 2.0 Hz, H-2), ^13^C NMR (100 MHz, CD_3_OD): *δ* 114.4 (C-5), 116.3 (C-2), 121.7 (C-1), 122.5 (C-6), 144.7 (C-), 150.1 (C-, 168.9 (C-7).

#### Gallic acid (7)

Compound 7 was isolated as colorless needle. Its molecular formula of C_7_H_6_O_5_ was determined by HRESIMS ion peak at *m/z* 169.07 [M-H]^−^ (calcd for C_7_H_5_O_5_, 169.01). ^1^H NMR (400 MHz, C_5_D_5_N): *δ* 8.08 (2H*, s*, H-2, H-6), ^13^C NMR (100 MHz, C_5_D_5_N): *δ* 110.3 (C-2, C-6), 122.7 (C-1), 140.3 (C-4), 147.4 (C-3, C-5), 169.4 (C-7).

#### GP-α and α-glucosidase inhibition activity of isolated compounds

The isolated compounds from *B. javanica* seeds were tested for α-glucosidase and GP-α inhibition activity in vitro and results are summarized in Table 2. Compound 5 was found to be the most potent inhibitor of GP-α and α-glucosidase, and its GP-α activity (IC_50_ = 45.08 μM) was 10 times more potent than that of standard GP-α inhibitor caffeine (IC_50_ = 457.34 μM), and its α-glucosidase inhibitory activity (IC_50_ = 26.41 μM) was 5.5 times more potent than that of acarbose (IC_50_ = 145.83 μM). Compounds 4, 6, and 7 are phenolic derivatives of benzoic acid. Interestingly, it can be observed that the increased number of hydroxyl substituent on the skeleton of benzoic acid significantly increased potency of those compounds against GP-α and α-glucosidase. Compound 1, which is a direct structural analogue of para-hydroxybenzoic acid (4) without the methoxy group, showed little to no inhibition (22.91%) of GP-α at 12 mM, and did not inhibit α-glucosidase at the concentrations tested up to 6 mM, suggested that the methoxy functionality in compound 1 was influential on its biological activity. Compounds 2 and 3 exhibited very weak inhibitory activity against both GP-α (19.88% and 32.74%) at 5 mM and α-glucosidase (14.74% and 16.14%) at 2.5 mM.

**Table 2:** GP-α and α-glucosidase inhibition activities of isolated compounds from *B. javanica* seeds

Compounds	Glycogen phosphorylase α (IC_50_ = μM)	α-glucosidase(IC_50_ = μM)
Vanillic Acid	ND^1^	ND^1^
Bruceine D	ND^2^	ND^2^
Bruceine E	ND^2^	ND^2^
Para-hydroxybenzoic acid	357.88 ± 0.07	649.07 ± 0.29
Luteolin	45.08 ± 0.04	26.41 ± 0.04
Protocatechuic acid	297.37 ± 0.13	368.74 ± 0.13
Gallic acid	214.38 ± 0.12	277.04 ± 0.12
Acarbose	-	145.83 ± 0.03
Caffeine	457.34 ± 0.05	-

^1^ND: IC_50_ values were not determined at the concentrations below 6 mM (α-glucosidase) and 12 mM (GP-α)

^2^ND IC_50_ values were not determined at the concentrations below 2.5 mM (α-glucosidase) and 5 mM (GP-α)

### Effect of EAF in type 2 diabetic rats

#### Effect of EAF on fasting blood glucose levels

Antihyperglycemic activity of EA fraction from *B. javanica* seeds was evaluated on T2D rat model. As Table 3 show, FBG levels was significantly (*p* < 0.05) elevated in DC group compared to non-diabetic control group. Treatment with EAF (25 and 50 mg/kg b.w.) and GLI (10 mg/kg b.w.) in T2D rats, once a day for 28 days, caused significant reductions in FBG levels compared to initial day. The reduction of FBG levels for EAF (25 and 50 mg/kg b.w.) and GLI (10 mg/kg b.w.) treated T2D rats were 29.78, 44.77, and 45.44%, respectively, compared to initial day of the respective groups. There was no significant difference for FBG levels in NDC rats throughout the experimental period.

**Table 3:** Effects of EAF from *B. javanica* seeds on fasting blood glucose levels in T2D rats

Groups	Fasting blood glucose level (mmol/L)				
	0 Day	Day 7	Day 14	Day 21	Day 28
NDC	4.38 ± 0.28	4.47 ± 0.38	4.28 ± 0.23	4.23 ± 0.10	4.17 ± 0.12
DC	12.83 ± 0.95	13.95 ± 0.77	16.22 ± 1.19	19.33 ± 1.45	22.25 ± 1.97^a^
D + EAF25	13.43 ± 1.01	13.25 ± 1.09	12.13 ± 1.71	10.98 ± 1.56	9.43 ± 1.12^a^
D + EAF50	14.25 ± 0.93	12.65 ± 0.67	11.02 ± 0.59	8.82 ± 0.67	7.87 ± 1.15^a^
D + GLI	16.90 ± 1.63	17.63 ± 2.13	12.97 ± 0.72	11.83 ± 1.27	9.22 ± 0.65^a^

The results are expressed as mean ± SE (*n* = 6). *NDC* non-diabetic control, *DC* diabetic control, *D + EAF25* diabetic rats treated with ethyl acetate fraction 25 mg/kg b.w., *D + EAF50* diabetic rats treated with ethyl acetate fraction 50 mg/kg b.w., *D + GLI* diabetic rats treated with glibenclamide 10 mg/kg b.w. The results are considered significant when *p* < 0.05

^**a**^Compared with 0 day

#### Effect of EAF on body weights

The effect of EAF from *B. javanica* seeds and GLI on body weight in experimental rats was summarized in Table 4. During the four-week study period, rats in NDC group continued to gain weight by 33.26%, whereas DC group continuously loss weight (18.91%) due to STZ toxicity compared to the initial day. Treatment with EAF and GLI prevented weight loss in diabetic rats and there was no significant decrease in the body weight of animals after four weeks of treatment when compared with 0 day.

**Table 4:** Effects of EAF from *B. javanica* seeds on body weights in T2D rats

Groups	Body Weight (g)				
	0 Day	Day 7	Day 14	Day 21	Day 28
NDC	214.00 ± 10.78	236.83 ± 7.44	259.50 ± 7.67	272.17 ± 8.68	285.17 ± 8.81^a^
DC	232.67 ± 7.37	220.83 ± 7.64	215.33 ± 7.21	197.17 ± 9.70	188.67 ± 7.54^a^
D + EAF25	215.00 ± 9.86	207.00 ± 10.17	203.67 ± 10.43	198.17 ± 10.80	201.00 ± 9.86
D + EAF50	222.83 ± 10.88	218.83 ± 12.36	216.83 ± 12.73	216.67 ± 12.48	217.83 ± 12.00
D + GLI	211.00 ± 6.19	203.50 ± 8.90	205.83 ± 7.31	206.00 ± 6.64	209.33 ± 7.91

The results are expressed as mean ± SE (*n* = 6). *NDC* non-diabetic control, *DC* diabetic control, *D + EAF25* diabetic rats treated with ethyl acetate fraction 25 mg/kg b.w., *D + EAF50* diabetic rats treated with ethyl acetate fraction 50 mg/kg b.w., *D + GLI* diabetic rats treated with glibenclamide 10 mg/kg b.w. The results are considered significant when *p* < 0.05

^**a**^Compared with 0 day

#### Effects of EAF on OGTT

**Fig. 3:**
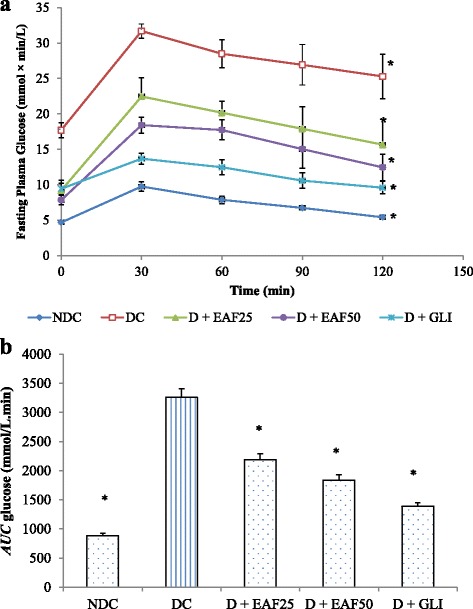
**a** Effects of EAF and GLI on oral glucose tolerance in experimental rats. NDC: non-diabetic control; DC: diabetic control, D + EAF25: diabetic rats treated with ethyl acetate fraction 25 mg/kg b.w.; D + EAF50: diabetic rats treated with ethyl acetate fraction 50 mg/kg b.w.; D + GLI: diabetic rats treated with glibenclamide 10 mg/kg b.w. The results represent the mean ± SE for 6 rats in each group. **b** Area under curve for glucose (*AUC*_glucose_) values for 0–120 min after glucose load. NDC: non-diabetic control; DC: diabetic control, D + EAF25: diabetic rats treated with ethyl acetate fraction 25 mg/kg b.w.; D + EAF50: diabetic rats treated with ethyl acetate fraction 50 mg/kg b.w.; D + GLI: diabetic rats treated with glibenclamide 10 mg/kg b.w. The results represent the mean ± SE for 6 rats in each group

Results in Fig. 3 (a and b) show the effect of EAF on T2D rat during OGTT. Blood glucose in all groups were elevated at 30 min time point post glucose load, and then gradually declined following hours. At 120 min, blood glucose levels were significantly (*p* <0.05) reduced to 30.26%, 32.46%, and 29.97% after treated with EAF (25 and 50 mg/kg) and GLI when compared to the values at 30 min (Fig. 3a). Further estimation of AUC for glucose at 0 to 120 min showed that degrease of glucose concentration was 32.82%, 43.54%, and 57.42% for EAF (25 and 50 mg/kg) and GLI groups, respectively when compared to DC group (Fig. 3b).

#### Effects of EAF on serum insulin levels

**Fig. 4:**
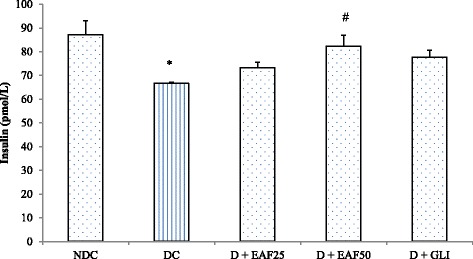
Effects of EAF on serum insulin in T2D rats. NDC: non-diabetic control; DC: diabetic control, D + EAF25: diabetic rats treated with ethyl acetate fraction 25 mg/kg b.w.; D + EAF50: diabetic rats treated with ethyl acetate fraction 50 mg/kg b.w.; D + GLI: diabetic rats treated with glibenclamide 10 mg/kg b.w. The results represent the mean ± SE for 6 rats in each group. Conversion factor: insulin (1 μg =174 pmol). * compared with NDC group. #compared with DC group

**Fig. 5:**
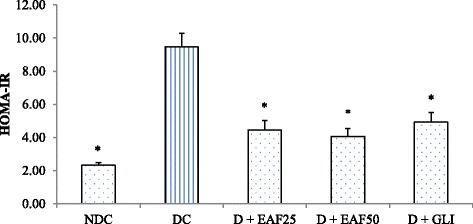
Effects of EAF on HOMA-IR index. NDC: non-diabetic control; DC: diabetic control, D + EAF25: diabetic rats treated with ethyl acetate fraction 25 mg/kg b.w.; D + EAF50: diabetic rats treated with ethyl acetate fraction 50 mg/kg b.w.; D + GLI: diabetic rats treated with glibenclamide 10 mg/kg b.w. The results represent the mean ± SE (*n* = 6). *Compared with DC

As shown in Fig. 4, serum insulin level was decreased significantly (*p* < 0.05) in the diabetic control group compared to non-diabetic control group. Treatment with EAF at 50 mg/kg dose exhibited significant (*p* < 0.05) increase of serum insulin by 23.7% compared to diabetic control group. Treatment with EAF at 25 mg/kg dose and GLI increased serum insulin levels by 10% and 16.5% respectively, and no significant (*p* > 0.05) changes in the serum insulin levels were observed in these groups compared to diabetic control group. Finally, EAF treatment at 50 mg/kg dose exhibited slight higher activity than that of glibenclamide. In addition, HOMA-IR levels in EAF treated groups were significantly improved by 53.0% and 57.1% compared to the diabetic control group (Fig. 5).

#### Effects of EAF on serum lipid profiles

As shown in Table 5, diabetic rats have shown a significant elevation in TG (51.6%), TC (22.6%), and LDL (40.2%), with a significant decrease in HDL (65.7%) when compared with NDC group. Treatment with EAF to diabetic rats at the dose of 25 mg/kg for 4 weeks significantly reduced serum TG, TC, and LDL levels by 47.31%, 48.39%, and 49.43%, while treated with EAF at the dose of 50 mg/kg, there was significant reduction of serum TG (48.39%), TC (47.10%), and LDL (55.17%) levels respectively as compared with DC group. However, EAF (25 and 50 mg/kg) exhibited significant low TC levels, where values of TC was 33.33% and 31.67% lower than NDC group, respectively. Additionally, serum HDL level was attenuated significantly (*p* < 0.05) in DC rats, and it was restored near to normal levels (NDC) after treated with EAF (50 mg/kg). Treatment with EAF at the dose of 25 mg/kg increased HDL level, but unable to normalize it (*p* > 0.05) when compared with NDC group. By comparison, GLI improved serum lipid profiles in diabetic rats, and there was no significant (*p* > 0.05) changes in serum TG, TC, HDL, and LDL levels when compared with NDC group.

**Table 5:** Effects of EAF from *B. javanica* seeds on serum lipid profiles in T2D rats

Groups	Serum lipid profiles (mmol/L)			
	TG	TC	HDL	LDL
Nondiabetic Control	0.45 ± 0.07	1.20 ± 0.08	1.37 ± 0.02	0.52 ± 0.03
Diabetic Control	0.93 ± 0.21^a^	1.55 ± 0.08^a^	0.47 ± 0.06^*^	0.87 ± 0.15^a^
Diabetic + EAF (25 mg/kg)	0.49 ± 0.01^b^	0.80 ± 0.09^c^	1.12 ± 0.05^*a^	0.44 ± 0.04^a^
Diabetic + EAF (50 mg/kg)	0.48 ± 0.04^b^	0.82 ± 0.06^c^	1.22 ± 0.04^a^	0.39 ± 0.03^a^
Diabetic + Glibenclamide (10 mg/kg)	0.52 ± 0.09^b^	1.12 ± 0.05^c^	1.31 ± 0.08^a^	0.59 ± 0.04^a^

*TG* triglyceride, *TC* total cholesterol, *HDL* high-density lipoprotein, *LDL* low-density lipoprotein. The results are presented the mean ± SE for 6 rats in each group

^a^Compared with NDC, ^b^ Compared with DC, ^c^ Compared with DC and NDC

#### Effects of EAF on renal and liver functions

As Table 6 shows, liver enzymes (ALP, ALT, and AST) and urea were increased significantly (*p* < 0.05) in DC group compared to the NDC group. Treatment with EAF (50 mg/kg) and GLI significantly reversed these alterations and there were no significant difference in the levels of liver enzymes and urea when compared with NDC group. Treatment with EAF (25 mg/kg) did not reduce ALT level to normal when compared with NDC group. The creatinine levels were not significantly (*p* > 0.05) altered in STZ (DC), EAF, and as well as GLI treated groups in comparison with NDC group.

**Table 6:** Effects of EAF from *B. javanica* seeds on liver and renal function markers in T2D rats

Groups	ALP(U/L)	ALT (U/L)	AST (U/L)	Urea (mmol/L)	Creatinine (μmol/L)
NDC	141.33 ± 16.11	45.50 ± 2.45	129.17 ± 8.93	6.23 ± 0.64	29.17 ± 1.01
DC	319.33 ± 10.99^a^	172.67 ± 15.09^a^	290.00 ± 11.55^a^	18.98 ± 2.73^a^	35.00 ± 2.11
D + EAF25	179.83 ± 19.83	81.17 ± 7.06^a^	159.50 ± 15.72	11.22 ± 1.27	30.50 ± 3.56
D + EAF50	159.17 ± 24.03	60.67 ± 3.35	145.33 ± 15.64	9.52 ± 1.52	27.50 ± 1.12
D + GLI	146.67 ± 10.50	58.17 ± 4.52	138.83 ± 8.33	10.37 ± 1.80	29.00 ± 0.93

*ALP* alkaline phosphatase, *ALT* alanine aminotransferase, *AST* aspartate aminotransferase. The results are presented the mean ± SE for 6 rats in each group. *NDC* non-diabetic control, *DC* diabetic control, *D + EAF25* diabetic rats treated with ethyl acetate fraction 25 mg/kg b.w., *D + EAF50* diabetic rats treated with ethyl acetate fraction 50 mg/kg b.w., *D + GLI* diabetic rats treated with glibenclamide 10 mg/kg b.w. The results are considered significant when *p* < 0.05

^a^Compared with NDC

#### Effects of EAF on MDA and GSH levels

**Fig. 6:**
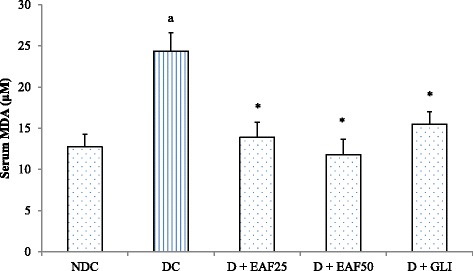
Effects of EAF on MDA levels in T2D rats. NDC: non-diabetic control; DC: diabetic control, D + EAF25: diabetic rats treated with ethyl acetate fraction 25 mg/kg b.w.; D + EAF50: diabetic rats treated with ethyl acetate fraction 50 mg/kg b.w.; D + GLI: diabetic rats treated with glibenclamide 10 mg/kg b.w. The results are considered significant when *p* < 0.05. ^a^Compared with NDC, *Compared with DC

**Fig. 7:**
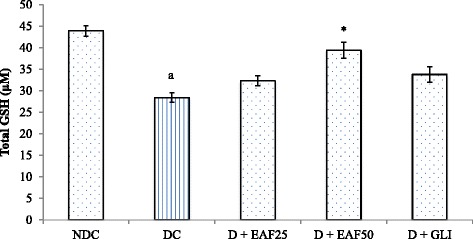
Effects of EAF on GSH levels in T2D rats. NDC: non-diabetic control; DC: diabetic control, D + EAF25: diabetic rats treated with ethyl acetate fraction 25 mg/kg b.w.; D + EAF50: diabetic rats treated with ethyl acetate fraction 50 mg/kg b.w.; D + GLI: diabetic rats treated with glibenclamide 10 mg/kg b.w. The results are considered significant when *p* < 0.05. ^a^Compared with NDC, *Compared with DC

Diabetic rats (DC) showed significant increase in serum MDA level compared to NDC group (Fig. 6). Treatment with EAF (25 and 50 mg/kg) resulted in significant (*p* < 0.05) reduction of serum MDA levels by 42.94% and 51.56% respectively as compared to the DC group, and the values of MDA in these groups were reversed to nearly normal as compared to the NDC group (*p* > 0.05). The GSH level was significantly decreased in the serum of DC group compared to NDC group (Fig. 7). Significant increase in serum GSH level was observed in EAF group treated with higher dose (50 mg/kg) and increase of GSH level was 38.70% compared with DC group. The lower dose (EAF at 25 mg/kg) increased serum GSH level by 13.80% compared to DC group, respectively. Treatment with GLI exhibited a significant reduction of about 36.45% of MDA level, but did not significantly increase GSH level and there was about 18.94% increase of serum GSH level when compared with DC group. By comparison, more effective changes in both MDA and GSH activity were observed with EAF treatment than with that of glibenclamide.

#### Effects of EAF on serum cytokine levels

The serum pro-inflammatory cytokine (TNFα, IL-6, and IL-1β) levels were significantly increased in DC rats compared with NDC (Table 7). However, these values were significantly (*p* < 0.05) reduced and restored near to normal level in diabetic rats treated with EAF (25 and 50 mg/kg), indicating that EAF effectively reduced the levels of proinflammatory cytokines in NA-STZ induced T2D rats

**Table 7:** Effects of EAF on serum cytokines and liver glycogen contents in T2D rats

Groups	TNF-α (pg/ml)	IL-6 (pg/ml)	IL-1β (pg/ml)	LG (mg/g tissue)
NDC	28.93 ± 6.48	89.17 ± 3.60	475.97 ± 20.64	15.57 ± 2.63
DC	122.60 ± 11.93^a^	171.67 ± 8.33^a^	1041.73 ± 43.71^a^	6.26 ± 0.67^a^
D + EAF25	76.77 ± 12.65^b^	116.94 ± 7.34^b^	839.76 ± 25.63^b^	10.11 ± 1.50
D + EAF50	59.10 ± 10.71^b^	97.78 ± 5.59^b^	590.97 ± 30.80^b^	12.67 ± 1.83
D + GLI	70.10 ± 4.00^b^	123.06 ± 10.18^b^	570.67 ± 31.71^b^	11.55 ± 1.82

*TNFα* tumor necrosis factor-alpha, *IL-6* interleukin-6, *IL-1β* interleukin-1β, *LG* liver glycogen, *NDC* non-diabetic control, *DC* diabetic control, *D + EAF25* diabetic rats treated with ethyl acetate fraction 25 mg/kg b.w., *D + EAF50* diabetic rats treated with ethyl acetate fraction 50 mg/kg b.w., *D + GLI* diabetic rats treated with glibenclamide 10 mg/kg b.w. Results are considered significant when *p* < 0.05

^a^Compared with NDC, ^b^Compared with DC

#### Effect of EAF on liver glycogen contents

Glycogen levels in the liver were exhausted significantly (*p* < 0.05) in NA-STZ treated rats (DC) and almost 60% of glycogen was lost when compared with NDC. Treatment with EAF and GLI increased hepatic glycogen accumulation, exhibiting values of glycogen similar to those of NDC group (Table 7).

## Discussion

*Brucea javanica* (L.) Merr is a well-known medicinal plant originated from tropical Asia. It is extensively used for the treatment of various diseases including diabetes in traditional medicine system among indigenous people in Malaya peninsula [19]. However, chemical entities responsible for potential inhibitory effect of this plant against GP-α and α-glucosidase enzymes are yet to be recognized. Results from the present study demonstrated that EAF of *B. javanica* seeds exhibited significant concentration-dependent inhibition on both GP-α and α-glucosidase enzymes activity in vitro compared to *n*-hexane, chloroform, and water fractions indicating that compounds in EAF could be involved in activities of carbohydrate and glycogen metabolism. Hence, based on in vitro assay, EAF was selected for further study.

In this study, T2D was developed in SD rats after NA-STZ treatment, as characterized by hyperglycemia compared to untreated rats (NDC). Treatment with EAF to diabetic rats significantly reduced blood glucose levels, increased glucose tolerance during OGTT, and increased insulin levels compared to untreated rats (DC). These results indicated that EAF exert antihyperglycemic effect by inhibiting GP-α and α-glucosidase activity to enhance glycogen synthesis and slowdown digestion of carbohydrates, thereby improve regulation of glucose in diabetic condition.

The body weight lowering effect is often found to be associated with diabetic conditions. It was observed that during the experimental period, body weight of EAF treated rats were slightly decreased compared to initial date. To evaluate the decrease in body weight either caused by diabetes or EAF, markers for liver (ALP, ALT, and AST) and kidney (urea and creatinine) toxicity were measured. In T2D rats, serum ALP, ALT, AST, and urea contents were significantly elevated after NA-STZ treatment, but significantly decreased in EAF treated rats without alteration of creatinine levels, indicating that EAF did not cause any damage to the liver and kidney when treated up to 50 mg/kg b.w. Hyperlipidemia is one of the major factors linked with hyperglycemia due to insulin deficiency during diabetes and correlated with carbohydrate metabolism. Elevation of cholesterol and triglyceride levels in T2D demonstrates abnormal lipoprotein metabolism associated with hyperglycemia [25, 26]. As shown in Table 5, serum TG, TC, and LDL contents were significantly increased, HDL content was decreased when experimental rats were made relatively insulin deficiency by NA-STZ treatment, but this entire abnormal lipid metabolism was restored to normal after EAF treatment. Interestingly, the T2D rats treated with EAF have significantly reduced TC and LDL contents and bring it to significantly lower level than that of NDC suggesting that α-glucosidase and GP-α inhibitors, mainly due to the presence of luteolin in EAF, may inhibit protein synthesis causing a decrease in the synthesis of the LDL protein and prevent cholesterol accumulation in T2D rats. Therefore, this phenomenon implies that decrease in body weight in EAF treated rats was not directly related to STZ toxicity or toxic effect accumulated during experimental period, and the weight lost in EAF treated rats were most probably due to the hypolipidimic effect of EAF.

As shown in Table 7, The EAF caused a significant reduction of serum TNF-α, IL-6, and IL-1β levels elevated in NA-STZ treated rats. TNF-α was a pro-inflammatory cytokine to be associated with insulin resistance due to reduced tyrosine kinase activity [27]. TNF-α and IL-6 can cause insulin resistance by suppressing expression of the insulin receptor substrate −1 (IRS-1) and GLUT-4 though activation of NF-_*K*_B pathway [27, 28]. The IL-1β inhibit IRS-1 signaling to promote insulin resistance [29]. Therefore, the significant reduction of TNF-α, IL-6, and IL-1β by EAF treatment can be explained as an outcome of its beneficial anti-inflammatory effect.

It has been reported that hyperglycemia and/or reduced antioxidant capacity of the body in diabetic condition result in elevation of ROS and RNS and consequently increased oxidative stress [30, 31]. To evaluate the effect of EAF to suppress oxidative stress, serum GSH and MDA levels were measured. Significant decrease of GSH and elevation of MDA contents were observed in NA-STZ treated rats. Treatment with EAF had reserved activity of GSH approaching control level resulted in significant decrease in lipid peroxidation product MDA indicated that EAF abrogate oxidative stress by improving antioxidant mechanism in T2D rats.

The ability of liver to store glycogen is impaired due to lack of insulin or insulin resistance. It is often linked with enhanced activity of glycogen phosphorylase to improve glycolysis, ultimately, causes hyperglycemia [32, 33]. To evaluate effect of EAF on hepatic glycogen metabolism, glycogen contents in liver of treated and untreated rats were measured. As shown in table 7, hepatic glycogen content was decreased with the decreased insulin content in NA-STZ treated rats (DC), and It was further restored due to EAF treatment indicating that antihyperglycemic effect of EAF may be due to the enhanced hepatic glycogen metabolism and improved insulin secretion from pancreatic β-cell in T2D rats.

In normal condition, starch was broken down to glucose by α-glucosidase after meals and utilized by cells as an energy source [34]. Glucose was also generated by glycolysis between meals to maintain blood glucose homeostasis, and or stored as glycogen in the liver after meals by glycogenesis in response to elevated glucose concentration and neuroendocrine signals. Both glycolysis and glycogenesis are mediated by activities of GP-α activated by insulin [35]. Hence, the inhibitors of these enzymes are vital molecular therapeutic targets for controlling hyperglycemia associated with T2D and its related complications. In present study, bioactivity-guided separation using various column chromatography methods have been adapted and led to isolated seven compounds from EAF of *B. javanica* seeds and all of the isolated compounds were tested for GP-α and α-glucosidase inhibition activity. As shown in table 2, luteolin (5) was identified as the most potent inhibitor of GP-α and α-glucosidase enzymes followed by para-hydroxybenzoic acid (4), protocatechuic acid (6), and gallic acid (7). The vanillic acid (1) is ineffective to both GP-α and α-glucosidase due to the presence of methoxy group in its skeleton. Bruceine D (2) and bruceine E (3) did not show any GP-α and α-glucosidase inhibition activity indicating that the antidiabetic effect reported on these compounds [19] may involve other mechanistic pathway. The literature revealed that luteolin inhibit α-glucosidase activity in vitro [36], *para*-hydroxybenzoic acid increased hepatic glycogen content [37], protocatechuic acid prevented inflammation and improved lipid metabolism by enhancing antioxidant mechanism in diabetic mice [38], gallic acid exhibited antidiabetic effect by increasing insulin secretion from pancreatic β-cell [39, 40], prevented oxidative stress by reducing MDA content through enhancing antioxidant enzyme activity [41], prevented proinflammatory cytokine generation [42] in diabetic rats. All these reports further support on the findings on the compounds found in EAF from *B. javanica* seeds.

## Conclusion

The EAF showed antihyperglycemic and antioxidant potential, and reduced the levels of pro-inflammatory cytokines in T2D rats. Our results indicated that the effects of EAF in T2D rats may be due to the presence of luteolin acted as potential α-glucosidase and GP-α inhibitors. Furthermore, it could also be a result of synergistic effect of *para*-hydroxybenzoic acid, protocatechuic acid, and gallic acid existed in *B. javanica* seeds acting on several processes. However, possible synergistic effects of these compounds are yet to be evaluated in vivo.

## Abbreviations

ACN: Acetonitrile
ALP: Alkaline phosphatase
ALT: Alanine aminotransferase
AST: Aspartate aminotransferase
AUC: Area under the curve
DCM: Dichloromethane
EAF: Ethyl acetate fraction
GLI: Glibenclamide
GP-α: Glycogen phosphorylase α
GSH: Glutathione
HDL: High-density lipoprotein
IC50: The half maximal inhibitory concentration
IL-1β: Interleukin-1β
IL-6: Interleukin-6
LCMS: Liquid chromatography mass spectrometry
LDL: Low-density lipoprotein
LG: Liver glycogen
MDA: Malondialdehyde
NA: Nicotinamide
NMR: Nuclear magnetic resonance
OGTT: Oral glucose tolerance test
p-NPG: P-Nitrophenyl α-D-Glucoside
STZ: Streptozotocin
T2D: Type 2 diabetes
TC: Total cholesterol
TG: Triglyceride
TNFα: Tumor necrosis factor-alpha
SE: Standard error

## References

[1] Guariguata L (2014). Global estimates of diabetes prevalence for 2013 and projections for 2035. Diabetes Res Clin Pract.

[2] ADA (2015). Standards of Medical Care in Diabetes—2015: Summary of Revisions. Diabetes Care.

[3] Mogensen UM (2015). Metformin in combination with various insulin secretagogues in type 2 diabetes and associated risk of cardiovascular morbidity and mortality—A retrospective nationwide study. Diabetes Res Clin Pract.

[4] Aso Y, et al. Sitagliptin, a DPP-4 inhibitor, alters the subsets of circulating CD4+ T cells in patients with type 2 diabetes. Diabetes Research and Clinical Practice. 2015;110(3):250–6. 10.1016/j.diabres.2015.10.012 26508675

[5] Hamada Y (2013). The alpha-glucosidase inhibitor miglitol affects bile acid metabolism and ameliorates obesity and insulin resistance in diabetic mice. Metabolism.

[6] Bonner C, et al. Inhibition of the glucose transporter SGLT2 with dapagliflozin in pancreatic alpha cells triggers glucagon secretion. Nat Med. 2015;21:512–7. 10.1038/nm.3828 25894829

[7] Imai C (2014). Cotreatment with the α-glucosidase inhibitor miglitol and DPP-4 inhibitor sitagliptin improves glycemic control and reduces the expressions of CVD risk factors in type 2 diabetic Japanese patients. Metabolism.

[8] Braunstein S (2003). New developments in type 2 diabetes mellitus: Combination therapy with a thiazolidinedione. Clin Ther.

[9] Norwood P (2012). Safety of Exenatide Once Weekly in Patients With Type 2 Diabetes Mellitus Treated With a Thiazolidinedione Alone or in Combination With Metformin for 2 Years. Clin Ther.

[10] Meier JJ, Bhushan A, Butler PC (2006). The potential for stem cell therapy in diabetes. Pediatr Res.

[11] Auberval N (2015). Oxidative stress type influences the properties of antioxidants containing polyphenols in RINm5F beta cells. Evid Based Complement Alternat Med.

[12] Oyenihi OR, Brooks NL, Oguntibeju OO (2015). Effects of kolaviron on hepatic oxidative stress in streptozotocin induced diabetes. BMC Complement Altern Med.

[13] Porto ML (2015). Increased oxidative stress and apoptosis in peripheral blood mononuclear cells of fructose-fed rats. Toxicol in Vitro.

[14] Zahari A (2014). Antiplasmodial and Antioxidant Isoquinoline Alkaloids from Dehaasia longipedicellata. Planta Med.

[15] Ceriello A, Motz E (2004). Is oxidative stress the pathogenic mechanism underlying insulin resistance, diabetes, and cardiovascular disease? The common soil hypothesis revisited. Arterioscler Thromb Vasc Biol.

[16] Pickup JC (2004). Inflammation and activated innate immunity in the pathogenesis of type 2 diabetes. Diabetes Care.

[17] Liu JH (2009). Chemical constituents of plants from the genus Brucea. Chem Biodivers.

[18] Lau S, Lin Z, Leung P (2010). Role of reactive oxygen species in brucein D-mediated p38-mitogen-activated protein kinase and nuclear factor-κB signalling pathways in human pancreatic adenocarcinoma cells. Br J Cancer.

[19] NoorShahida A, Wong TW, Choo CY (2009). Hypoglycemic effect of quassinoids from Brucea javanica (L.) Merr (Simaroubaceae) seeds. J Ethnopharmacol.

[20] Ablat A (2014). Evaluation of Antidiabetic and Antioxidant Properties of Brucea javanica Seed. Sci World J.

[21] Lordan S (2013). The α-amylase and α-glucosidase inhibitory effects of Irish seaweed extracts. Food Chem.

[22] Cuevas-Juárez E (2014). Antioxidant and α-glucosidase inhibitory properties of soluble melanins from the fruits of Vitex mollis Kunth, Randia echinocarpa Sessé et Mociño and Crescentia alata Kunth. J Funct Foods.

[23] Arya A (2012). Anti-diabetic effects of Centratherum anthelminticum seeds methanolic fraction on pancreatic cells, β-TC6 and its alleviating role in type 2 diabetic rats. J Ethnopharmacol.

[24] Chang C-W (2015). Effects of combined extract of cocoa, coffee, green tea and garcinia on lipid profiles, glycaemic markers and inflammatory responses in hamsters. BMC Complement Altern Med.

[25] Grundy M, P.S.M (1998). Hypertriglyceridemia, Atherogenic Dyslipidemia, and the Metabolic Syndrome. Am J Cardiol.

[26] Abbate SL, Brunzell JD (1990). Pathophysiology of hyperlipidemia in diabetes mellitus. J Cardiovasc Pharmacol.

[27] Rui L (2001). Insulin/IGF-1 and TNF-α stimulate phosphorylation of IRS-1 at inhibitory Ser307 via distinct pathways. J Clin Investig.

[28] Gratas-Delamarche A (2014). Physical inactivity, insulin resistance, and the oxidative-inflammatory loop. Free Radic Res.

[29] Aye ILMH, Jansson T, Powell TL (2013). Interleukin-1β inhibits insulin signaling and prevents insulin-stimulated system A amino acid transport in primary human trophoblasts. Mol Cell Endocrinol.

[30] Hatem E (2014). Glutathione is essential to preserve nuclear function and cell survival under oxidative stress. Free Radic Biol Med.

[31] Evans JL (2003). Are Oxidative Stress − Activated Signaling Pathways Mediators of Insulin Resistance and β-Cell Dysfunction?. Diabetes.

[32] Grekinis D, Reimann EM, Schlender KK (1995). Phosphorylation and inactivation of rat heart glycogen synthase by cAMP-dependent and cAMP-independent protein kinases. Int J Biochem Cell Biol.

[33] Rajas F, Labrune P, Mithieux G (2013). Glycogen storage disease type 1 and diabetes: Learning by comparing and contrasting the two disorders. Diabetes Metab.

[34] McDougall GJ (2005). Different Polyphenolic Components of Soft Fruits Inhibit α-Amylase and α-Glucosidase. J Agric Food Chem.

[35] Agius, L., Role of glycogen phosphorylase in liver glycogen metabolism. Mol Asp Med, 2015. 10.1016/j.mam.2015.09.002 26519772

[36] Yan J (2014). α-Glucosidase inhibition by luteolin: Kinetics, interaction and molecular docking. Int J Biol Macromol.

[37] Peungvicha P (1998). 4-Hydroxybenzoic acid: a hypoglycemic constituent of aqueous extract of Pandanus odorus root. J Ethnopharmacol.

[38] Lende AB (2011). Anti-inflammatory and analgesic activity of protocatechuic acid in rats and mice. Inflammopharmacology.

[39] Latha RCR, Daisy P (2011). Insulin-secretagogue, antihyperlipidemic and other protective effects of gallic acid isolated from Terminalia bellerica Roxb. in streptozotocin-induced diabetic rats. Chem Biol Interact.

[40] Gandhi GR (2014). Gallic acid attenuates high-fat diet fed-streptozotocin-induced insulin resistance via partial agonism of PPARγ in experimental type 2 diabetic rats and enhances glucose uptake through translocation and activation of GLUT4 in PI3K/p-Akt signaling pathway. Eur J Pharmacol.

[41] Punithavathi VR (2011). Antihyperglycaemic, antilipid peroxidative and antioxidant effects of gallic acid on streptozotocin induced diabetic Wistar rats. Eur J Pharmacol.

[42] Ahad A (2015). Gallic acid ameliorates renal functions by inhibiting the activation of p38 MAPK in experimentally induced type 2 diabetic rats and cultured rat proximal tubular epithelial cells. Chem Biol Interact.

